# Assessment of the utility and performance of SORMAS in low- and middle-income countries: a scoping review protocol

**DOI:** 10.1186/s13643-025-02980-3

**Published:** 2025-12-05

**Authors:** Sylvia Amartekai Danso, Fortress Yayra Aku, Sam Newton, Wilm Quentin, Daniel Opoku

**Affiliations:** 1 School of Public Health, Kwame Nkrumah University of Science and Technology, Kumasi, Ghana; 2 German West African Centre for Global Health and Pandemic Prevention, Kumasi, Ghana; 3 Department of Epidemiology and Biostatistics, Fred N. Binka School of Public Health, University of Health and Allied Sciences, Hohoe Campus, Hohoe, Ghana; 4 Chair of Planetary and Public Health, University of Bayreuth, Bayreuth, Germany; 5 Department of Health Care Management, Technische Universität Berlin, Berlin, Germany

**Keywords:** Public health surveillance, Digital health, SORMAS

## Abstract

**Introduction:**

The Surveillance Outbreak Response Management and Analysis System (SORMAS) is an open source digital tool created to enhance real time surveillance and outbreak response especially in resource poor settings like low- and middle-income countries (LMICs). Even though the tool has been deployed in several countries, there is no comprehensive review of the available research reporting its implementation and the evidence on utility and performance as experienced by countries. This review aims to systematically map out available evidence to assess the utility and performance of SORMAS across LMICs systematically.

**Methods and analysis:**

The review will follow the Joanna Briggs Institute (JBI) approach to scoping review. Pubmed, Scopus, Web of Science, Google Scholar and Google will be searched as well as relevant grey literature sources. Studies will be included if they described or evaluated the adoption, implementation, utility or functionality of SORMAS in any LMIC. Studies will be excluded if they just mentioned SORMAS as a digital surveillance tool but did not describe or evaluate it. Titles, abstracts and full text screening will be done. Results will be summarized using descriptive statistics and thematic analysis. Tables and other visual tools will also be used to present the results.

**Discussion:**

The findings of this review will serve as the foundation for understanding how SORMAS is being used in LMICs and the specified context of implementation, as well as the reported performance. Gaps in implementation and research will also be highlighted. These findings will have important implications for policymakers, implementers, and researchers by highlighting best practices, areas needing capacity strengthening, and gaps requiring further investigation.

**Systematic review registration:**

10.17605/OSF.IO/DV8RJ.

**Supplementary Information:**

The online version contains supplementary material available at 10.1186/s13643-025-02980-3.

## Background

In the past few decades, digital health interventions specifically Mobile health (mHealth) interventions have shown promise in improving health outcomes, increasing access to care, and strengthening health systems, especially in low- and middle-income countries (LMICs) [1, 2]. LMICs face a persistently high burden of epidemic-prone diseases and endure frequent outbreaks worsened by socioeconomic and environmental vulnerabilities [3]. Yet in the field of public health surveillance, disease surveillance systems remain largely paper-based resulting in delayed reporting, incomplete data, and limited responsiveness [4]. The rapid proliferation of mobile technology in these settings offers a unique opportunity to address these longstanding challenges by reducing time to outbreak detection, improving transparency to outbreak information and quick analysis [5, 6] particularly suited to such resource-constrained settings.

Among the growing number of mHealth innovations, the Surveillance Outbreak Response Management and Analysis System (SORMAS) has garnered attention for its potential to support real time surveillance and outbreak response and seamless stakeholder communication [7–9]. Despite its growing deployment, there remains limited consolidated evidence on how SORMAS is being used across LMICs and the extent to which it has demonstrated usefulness in diverse contexts. Understanding the degree and nature of SORMAS implementation can inform better implementation strategies, highlight success factors, and identify gaps requiring further investigation.

Scoping reviews are particularly well-suited for mapping the breadth and depth of evidence on complex or emerging topics [10]. This review aims to systematically explore and synthesize available literature on the utility and performance of SORMAS. By providing a comprehensive overview of the existing literature, this scoping review will contribute to a better understanding of the current landscape of mHealth-based health surveillance innovation in LMICs and offer insights to guide future research, policy, and practice related to the SORMAS intervention.

## Method and analysis

The Joanna Briggs Institute (JBI) manual for evidence synthesis [11] which is a modification of the Arksey and O’Malley framework [12] will be used as the framework for this scoping review. The review will be carried out following these steps: (1) determining the research question, (2) identifying relevant studies, (3) study selection, (4) data extraction and charting and (5) data analysis and reporting.

### Determining the research question

A preliminary search of Google Scholar and the website of the SORMAS foundation revealed that SORMAS-related research has mostly been at the country level with varied methodologies using primary data. Although an unpublished report [13] has partially reviewed SORMAS using desk review and in-depth interviews with stakeholders in an unpublished report, it is still considered grey literature with a limited scope that has not been subjected to peer review. Although that work provides some first insights into the application of SORMAS, it does not fully address the utility of SORMAS in LMICs. By using a more rigorous methodological framework, this current review will be updating the literature to reflect more recent advancements and encompassing a wider range of sources. By doing this, it seeks to offer a more methodical, open, and scholarly examination of SORMAS subjected to the peer review process. This scoping review will answer the primary question: What information is available on the utility and performance of SORMAS in LMICs? More specifically, the research questions are as follows:

1. At which administrative level is SORMAS being used and who are the main actors in LMICs?
2. What was the context of adoption and scale-up and what is the current use case of SORMAS at the country level in LMICs?
3. What are the barriers and facilitators of SORMAS adoption, including scale-up at the country level in LMICs?
4. What indicators have been used to assess performance of SORMAS?
5. What is the evidence of the performance of SORMAS in LMICs?
6. What are the existing gaps in SORMAS implementation and research?

### Identifying relevant studies

Using the research question as a basis, a preliminary search was carried out on PubMed and Google Scholar using key terms based on the JBI framework for search strategy development Population-Concept-Context (PCC).

Population: The population of interest for this review will be all stakeholders involved in SORMAS implementation including policy makers, surveillance officers, program managers, IT managers, and other healthcare professionals who are the target users and direct beneficiaries of the SORMAS tool within the broader health systems. Even though individual participants are not the main focus of this review, their data may be indirectly involved in the primary studies.

Concept: The Surveillance Outbreak Response Management and Analysis System (SORMAS) is the concept for this review.

Context: The review is focused on LMICs as defined by the World Bank [14] who are making use of SORMAS.

### Study selection

The literature search will be conducted across multiple databases, including PubMed, Google Scholar, Google, Scopus, and Web of Science. Grey literature including relevant technical and institutional reports will also be extracted from the websites of the World Health Organization (WHO) and the SORMAS foundation. The search terms will include these keywords and their MESH term: “surveillance outbreak response management and analysis system,” “SORMAS,” and “LMICs.” These terms will be combined using Boolean operators, resulting in the following search query: (“surveillance outbreak response management and analysis system” OR “SORMAS” AND (“LMICs” OR individually listed LMICs). The aim of this combination of keywords and Boolean logic will be to comprehensively gather studies directly related to the research focus. Search query will be adapted to the various databases and results from all included databases will be imported into the Mendeley reference manager. A draft search strategy has been included as supplementary file 1. After deduplication, title and abstract screening as well as full text screening will be done by two independent reviewers based on the inclusion and exclusion criteria. Non-consensus will be resolved by a third reviewer after consultation. The whole study selection process will be guided and documented according to the Preferred Reporting Items for Systematic Reviews and Meta-Analyses: Extension for Scoping Review Guidelines (PRISMA-ScR) flow diagram [15]. The PRISMA-P checklist has been provided as supplementary file 2.

### Eligibility criteria

***Inclusion criteria***

1. Studies on SORMAS implementation, adoption, effectiveness, or challenges [16].
2. Articles focusing on LMICs.
3. Peer-reviewed journal articles, reports, conference proceedings, and grey literature (e.g., government or institutional reports).

***Exclusion criteria***

1. Articles that refer to SORMAS but do not have a clear focus on the utility of the tool.
2. Studies based in high-income countries.
3. Studies that refer to surveillance tools other than SORMAS

### Data extraction and charting

Two reviewers will work independently in extracting data from selected studies using a custom-made extraction form that will be piloted before use. The extraction form will be modified during the pilot test by reviewing any discrepancies noted by the reviewers. Study findings will be extracted and organized according to the specific research questions under the research objectives. A tabular format will be developed in Microsoft Excel to summarize the evidence on the country, utility, actors involved, context of implementation, disease conditions and stage of implementation. Evidence on performance assessment will also be summarized taking into consideration study design, indicators assessed, and the results of these assessments. The definition of performance will be assessed from individual studies.

### Summarising and presentation of results

The PRISMA-flow diagram will be used to document the study selection process. The results of the scoping review will be reported using descriptive summaries and thematic narrative synthesis. Descriptive summaries of included studies will be created in a tabular format giving information on study locations (countries) and their corresponding study designs and data sources. Thematic narrative analysis will be used to summarize findings concerning all main areas of study objectives such as reported utility of SORMAS, implementation context, facilitators and barriers to implementation, performance indicators measured and reported outcomes. Under this section, findings will be categorized by country. Facilitators and barriers will be grouped into extrinsic factors (factors pertaining to the health system) and intrinsic (factors pertaining to SORMAS). Visual tools such as maps will also be used to support the presentation of the results.

In addition to these findings, gaps in research and evidence will be highlighted to inform future research and stakeholder engagements.

### Quality appraisal

In line with scoping review methodology, we will not conduct a formal risk of bias or quality assessment. The aim of this review is to map and synthesize existing evidence on the utility and performance of SORMAS at country level, regardless of study design or methodological quality. The study will focus on systematically charting study characteristics and extracting relevant findings to provide a comprehensive overview of the evidence base.

### Dissemination

The review protocol has been registered in the Open Science Framework (OSF) Registries (https://osf.io/dv8rj). This registration of this planned review has made both the protocol and methodological framework publicly accessible prior to the commencement of the review itself. This practice facilitates the incorporation of peer feedback and establishes an open, verifiable, and credible review process, thereby strengthening the overall transparency of the research. Review findings will be published in a peer-reviewed journal and presented at both local and international scientific platforms.

### Current status of the study

It is expected that the review will be completed by August 2025, with the database search having started in April 2025.

## Discussion

With the rise in digital health solutions especially in resource-poor settings and the increasing role they play in strengthening health systems, it is paramount that evidence is generated and synthesized to inform their adoption, scale-up, sustainability, and policy integration. Although primary research in some countries has reported on the implementation of SORMAS [7, 17], there is currently no review mapping out the broader landscape of this in LMICs. This review will fill that gap by presenting a comprehensive overview of the evidence on the utility and performance of SORMAS across LMICs. This will inform future adoption in similar settings and inform decision-making among stakeholders.

## Strengths and limitations of this study

**•**The methodological rigour of the study will ensure reproducibility and the selection of the most relevant articles.

•This review will put into perspective SORMAS implementation and utility in LMICs.

•A focus on the LMIC context will bring to the fore unique challenges and opportunities peculiar to this setting.

•The review will examine tangible and intangible benefits and outcomes of SORMAS, providing useful insights for other countries who are yet to implement or scale-up SORMAS.

**•**The review may be limited by the small number of eligible articles as the tool is relatively new in some settings and included studies may differ in methodology and outcome measures. This will be duly appraised and reported accordingly.

## Supplementary Information

Additional file 1. Search strategy

Additional file 2. PRISMA checklist

## Data Availability

Not applicable.

## Abbreviations

LMICs: Low and Middle-Income Countries
SORMAS: Surveillance Outbreak Response Management and Analysis System
WHO: World Health Organization
