# Supplementary material for: Assessment of the utility and performance of SORMAS in low- and middle-income countries: a scoping review protocol
Source: Syst Rev. 2025 Dec 5;15:5. doi: 10.1186/s13643-025-02980-3 (PMC12797772; doi:10.1186/s13643-025-02980-3)
Supplement: Supplementary file 1 — Additional file 1. Search strategy [file 13643_2025_2980_MOESM1_ESM.docx]

PUBMED

("SORMAS"[All Fields] OR “surveillance outbreak response management and analysis system”[All fields] OR (("epidemiology"[MeSH Subheading] OR "epidemiology"[All Fields] OR "surveillance"[All Fields] OR "epidemiology"[MeSH Terms] OR "surveilance"[All Fields] OR "surveillances"[All Fields] OR "surveilled"[All Fields] OR "surveillence"[All Fields]) AND ("disease outbreaks"[MeSH Terms] OR ("disease"[All Fields] AND "outbreaks"[All Fields]) OR "disease outbreaks"[All Fields] OR "outbreak"[All Fields] OR "epidemiology"[MeSH Subheading] OR "epidemiology"[All Fields] OR "outbreaks"[All Fields] OR "outbreak s"[All Fields]) AND ("response"[All Fields] OR "responses"[All Fields] OR "responsive"[All Fields] OR "responsiveness"[All Fields] OR "responsivenesses"[All Fields] OR "responsives"[All Fields] OR "responsivities"[All Fields] OR "responsivity"[All Fields]) AND ("manage"[All Fields] OR "managed"[All Fields] OR "management s"[All Fields] OR "managements"[All Fields] OR "manager"[All Fields] OR "manager s"[All Fields] OR "managers"[All Fields] OR "manages"[All Fields] OR "managing"[All Fields] OR "managment"[All Fields] OR "organization and administration"[MeSH Terms] OR ("organization"[All Fields] AND "administration"[All Fields]) OR "organization and administration"[All Fields] OR "management"[All Fields] OR "disease management"[MeSH Terms] OR ("disease"[All Fields] AND "management"[All Fields]) OR "disease management"[All Fields]) AND ("systems analysis"[MeSH Terms] OR ("systems"[All Fields] AND "analysis"[All Fields]) OR "systems analysis"[All Fields] OR ("analysis"[All Fields] AND "system"[All Fields]) OR "analysis system"[All Fields]))) AND ("developing countries"[MeSH Terms] OR ("developing"[All Fields] AND "countries"[All Fields]) OR "developing countries"[All Fields] OR "lmics"[All Fields] OR "lmic s"[All Fields] OR "LMIC"[All Fields] OR ("afghanistan"[MeSH Terms] OR "afghanistan"[All Fields] OR "afghanistan s"[All Fields] OR ("albania"[MeSH Terms] OR "albania"[All Fields]) OR ("algeria"[MeSH Terms] OR "algeria"[All Fields]) OR ("angola"[MeSH Terms] OR "angola"[All Fields] OR "angola s"[All Fields]) OR ("argentina"[MeSH Terms] OR "argentina"[All Fields] OR "argentina s"[All Fields] OR "argentinae"[All Fields]) OR ("armenia"[MeSH Terms] OR "armenia"[All Fields]) OR ("azerbaijan"[MeSH Terms] OR "azerbaijan"[All Fields]) OR ("bangladesh"[MeSH Terms] OR "bangladesh"[All Fields] OR "bangladesh s"[All Fields]) OR ("republic of belarus"[MeSH Terms] OR ("republic"[All Fields] AND "belarus"[All Fields]) OR "republic of belarus"[All Fields] OR "belarus"[All Fields]) OR ("belize"[MeSH Terms] OR "belize"[All Fields]) OR ("benin"[MeSH Terms] OR "benin"[All Fields] OR "benin s"[All Fields]) OR ("bhutan"[MeSH Terms] OR "bhutan"[All Fields] OR "bhutan s"[All Fields]) OR ("bolivia"[MeSH Terms] OR "bolivia"[All Fields]) OR ("bosnia and herzegovina"[MeSH Terms] OR ("bosnia"[All Fields] AND "herzegovina"[All Fields]) OR "bosnia and herzegovina"[All Fields]) OR ("botswana"[MeSH Terms] OR "botswana"[All Fields] OR "botswana s"[All Fields]) OR ("brazil"[MeSH Terms] OR "brazil"[All Fields] OR "brazil s"[All Fields] OR "brazils"[All Fields]) OR ("bulgaria"[MeSH Terms] OR "bulgaria"[All Fields]) OR ("burkina faso"[MeSH Terms] OR ("burkina"[All Fields] AND "faso"[All Fields]) OR "burkina faso"[All Fields]) OR ("burundi"[MeSH Terms] OR "burundi"[All Fields]) OR ("cabo verde"[MeSH Terms] OR ("cabo"[All Fields] AND "verde"[All Fields]) OR "cabo verde"[All Fields]) OR ("cambodia"[MeSH Terms] OR "cambodia"[All Fields] OR "cambodia s"[All Fields]) OR ("cameroon"[MeSH Terms] OR "cameroon"[All Fields] OR "cameroons"[All Fields] OR "cameroon s"[All Fields]) OR ("central african republic"[MeSH Terms] OR ("central"[All Fields] AND "african"[All Fields] AND "republic"[All Fields]) OR "central african republic"[All Fields]) OR ("chad"[MeSH Terms] OR "chad"[All Fields]) OR ("china"[MeSH Terms] OR "china"[All Fields] OR "china s"[All Fields] OR "chinas"[All Fields]) OR ("colombia"[MeSH Terms] OR "colombia"[All Fields] OR "colombia s"[All Fields]) OR ("comoros"[MeSH Terms] OR "comoros"[All Fields] OR "comoro"[All Fields]) OR (("congo"[MeSH Terms] OR "congo"[All Fields]) AND "dem"[All Fields] AND "rep"[All Fields]) OR (("congo"[MeSH Terms] OR "congo"[All Fields]) AND "rep"[All Fields]) OR ("costa rica"[MeSH Terms] OR ("costa"[All Fields] AND "rica"[All Fields]) OR "costa rica"[All Fields]) OR ("cote d ivoire"[MeSH Terms] OR ("cote"[All Fields] AND "d ivoire"[All Fields]) OR "cote d ivoire"[All Fields]) OR ("cuba"[MeSH Terms] OR "cuba"[All Fields]) OR ("djibouti"[MeSH Terms] OR "djibouti"[All Fields]) OR ("dominica"[MeSH Terms] OR "dominica"[All Fields]) OR ("dominican republic"[MeSH Terms] OR ("dominican"[All Fields] AND "republic"[All Fields]) OR "dominican republic"[All Fields]) OR ("ecuador"[MeSH Terms] OR "ecuador"[All Fields] OR "ecuador s"[All Fields]) OR (("egypt"[MeSH Terms] OR "egypt"[All Fields] OR "egypt s"[All Fields]) AND ("arabs"[MeSH Terms] OR "arabs"[All Fields] OR "arab"[All Fields]) AND "rep"[All Fields]) OR ("el salvador"[MeSH Terms] OR ("el"[All Fields] AND "salvador"[All Fields]) OR "el salvador"[All Fields]) OR ("equatorial guinea"[MeSH Terms] OR ("equatorial"[All Fields] AND "guinea"[All Fields]) OR "equatorial guinea"[All Fields]) OR ("eritrea"[MeSH Terms] OR "eritrea"[All Fields]) OR ("eswatini"[MeSH Terms] OR "eswatini"[All Fields]) OR ("ethiopia"[MeSH Terms] OR "ethiopia"[All Fields] OR "ethiopia s"[All Fields]) OR ("fiji"[MeSH Terms] OR "fiji"[All Fields]) OR ("gabon"[MeSH Terms] OR "gabon"[All Fields]) OR ("gambia"[MeSH Terms] OR "gambia"[All Fields] OR "gambia s"[All Fields]) OR ("georgia"[MeSH Terms] OR "georgia"[All Fields] OR "georgia republic"[MeSH Terms] OR ("georgia"[All Fields] AND "republic"[All Fields]) OR "georgia republic"[All Fields] OR "georgia s"[All Fields]) OR ("ghana"[MeSH Terms] OR "ghana"[All Fields] OR "ghana s"[All Fields]) OR ("grenada"[MeSH Terms] OR "grenada"[All Fields]) OR ("guatemala"[MeSH Terms] OR "guatemala"[All Fields] OR "guatemala s"[All Fields]) OR ("guinea"[MeSH Terms] OR "guinea"[All Fields] OR "guinea s"[All Fields] OR "guineas"[All Fields]) OR ("guinea bissau"[MeSH Terms] OR "guinea bissau"[All Fields] OR ("guinea"[All Fields] AND "bissau"[All Fields]) OR "guinea bissau"[All Fields]) OR ("guyana"[MeSH Terms] OR "guyana"[All Fields]) OR ("haiti"[MeSH Terms] OR "haiti"[All Fields] OR "haiti s"[All Fields]) OR ("honduras"[MeSH Terms] OR "honduras"[All Fields]) OR ("india"[MeSH Terms] OR "india"[All Fields] OR "india s"[All Fields] OR "indias"[All Fields]) OR ("indonesia"[MeSH Terms] OR "indonesia"[All Fields] OR "indonesia s"[All Fields] OR "indonesias"[All Fields]) OR (("iran"[MeSH Terms] OR "iran"[All Fields]) AND ("islam"[MeSH Terms] OR "islam"[All Fields] OR "islamic"[All Fields] OR "islam s"[All Fields] OR "islamism"[All Fields]) AND "rep"[All Fields]) OR ("iraq"[MeSH Terms] OR "iraq"[All Fields]) OR ("jamaica"[MeSH Terms] OR "jamaica"[All Fields] OR "jamaica s"[All Fields]) OR ("jordan"[MeSH Terms] OR "jordan"[All Fields]) OR ("kazakhstan"[MeSH Terms] OR "kazakhstan"[All Fields] OR "kazakhstan s"[All Fields]) OR ("kenya"[MeSH Terms] OR "kenya"[All Fields] OR "kenya s"[All Fields]) OR ("kiribati"[MeSH Terms] OR "kiribati"[All Fields]) OR (("korea"[MeSH Terms] OR "korea"[All Fields] OR "korea s"[All Fields] OR "koreas"[All Fields]) AND "dem"[All Fields] AND ("people s"[All Fields] OR "peopled"[All Fields] OR "peopling"[All Fields] OR "persons"[MeSH Terms] OR "persons"[All Fields] OR "people"[All Fields] OR "peoples"[All Fields]) AND "rep"[All Fields]) OR ("kosovo"[MeSH Terms] OR "kosovo"[All Fields] OR "kosovo s"[All Fields]) OR ("kyrgyzstan"[MeSH Terms] OR "kyrgyzstan"[All Fields] OR ("kyrgyz"[All Fields] AND "republic"[All Fields]) OR "kyrgyz republic"[All Fields]) OR ("Lao"[All Fields] AND "PDR"[All Fields]) OR ("lebanon"[MeSH Terms] OR "lebanon"[All Fields] OR "lebanon s"[All Fields]) OR ("lesotho"[MeSH Terms] OR "lesotho"[All Fields] OR "lesotho s"[All Fields]) OR ("liberia"[MeSH Terms] OR "liberia"[All Fields] OR "liberia s"[All Fields]) OR ("libya"[MeSH Terms] OR "libya"[All Fields]) OR ("madagascar"[MeSH Terms] OR "madagascar"[All Fields] OR "madagascar s"[All Fields]) OR ("malawi"[MeSH Terms] OR "malawi"[All Fields] OR "malawi s"[All Fields]) OR ("malaysia"[MeSH Terms] OR "malaysia"[All Fields] OR "malaysia s"[All Fields]) OR ("maldive"[All Fields] OR "maldives"[MeSH Terms] OR "maldives"[All Fields]) OR ("mali"[MeSH Terms] OR "mali"[All Fields]) OR ("micronesia"[MeSH Terms] OR "micronesia"[All Fields] OR ("marshall"[All Fields] AND "islands"[All Fields]) OR "marshall islands"[All Fields]) OR ("mauritania"[MeSH Terms] OR "mauritania"[All Fields]) OR ("mauritius"[MeSH Terms] OR "mauritius"[All Fields]) OR ("mexico"[MeSH Terms] OR "mexico"[All Fields] OR "mexico s"[All Fields] OR "mexicos"[All Fields]) OR (("micronesia"[MeSH Terms] OR "micronesia"[All Fields]) AND "fed"[All Fields] AND "sts"[All Fields]) OR ("moldova"[MeSH Terms] OR "moldova"[All Fields]) OR ("mongolia"[MeSH Terms] OR "mongolia"[All Fields] OR "mongolia s"[All Fields]) OR ("montenegro"[MeSH Terms] OR "montenegro"[All Fields]) OR ("morocco"[MeSH Terms] OR "morocco"[All Fields]) OR ("mozambique"[MeSH Terms] OR "mozambique"[All Fields] OR "mozambique s"[All Fields]) OR ("myanmar"[MeSH Terms] OR "myanmar"[All Fields] OR "myanmar s"[All Fields] OR "myanmars"[All Fields]) OR ("namibia"[MeSH Terms] OR "namibia"[All Fields] OR "namibia s"[All Fields]) OR ("nepal"[MeSH Terms] OR "nepal"[All Fields] OR "nepal s"[All Fields]) OR ("nicaragua"[MeSH Terms] OR "nicaragua"[All Fields] OR "nicaragua s"[All Fields]) OR ("niger"[MeSH Terms] OR "niger"[All Fields]) OR ("nigeria"[MeSH Terms] OR "nigeria"[All Fields] OR "nigeria s"[All Fields]) OR ("republic of north macedonia"[MeSH Terms] OR ("republic"[All Fields] AND "north"[All Fields] AND "macedonia"[All Fields]) OR "republic of north macedonia"[All Fields] OR ("north"[All Fields] AND "macedonia"[All Fields]) OR "north macedonia"[All Fields]) OR ("pakistan"[MeSH Terms] OR "pakistan"[All Fields] OR "pakistan s"[All Fields]) OR ("palau"[MeSH Terms] OR "palau"[All Fields] OR "palau s"[All Fields]) OR ("panama"[MeSH Terms] OR "panama"[All Fields] OR "panama s"[All Fields]) OR ("papua new guinea"[MeSH Terms] OR ("papua"[All Fields] AND "new"[All Fields] AND "guinea"[All Fields]) OR "papua new guinea"[All Fields]) OR ("paraguai"[All Fields] OR "paraguay"[MeSH Terms] OR "paraguay"[All Fields]) OR ("peru"[MeSH Terms] OR "peru"[All Fields]) OR ("philippine"[All Fields] OR "philippines"[MeSH Terms] OR "philippines"[All Fields]) OR ("rwanda"[MeSH Terms] OR "rwanda"[All Fields] OR "rwanda s"[All Fields]) OR ("samoa"[MeSH Terms] OR "samoa"[All Fields] OR "samoas"[All Fields]) OR ("sao tome and principe"[MeSH Terms] OR ("sao"[All Fields] AND "tome"[All Fields] AND "principe"[All Fields]) OR "sao tome and principe"[All Fields]) OR ("senegal"[MeSH Terms] OR "senegal"[All Fields] OR "senegal s"[All Fields]) OR ("serbia"[MeSH Terms] OR "serbia"[All Fields]) OR ("sierra leone"[MeSH Terms] OR ("sierra"[All Fields] AND "leone"[All Fields]) OR "sierra leone"[All Fields]) OR ("melanesia"[MeSH Terms] OR "melanesia"[All Fields] OR ("solomon"[All Fields] AND "islands"[All Fields]) OR "solomon islands"[All Fields]) OR ("somalia"[MeSH Terms] OR "somalia"[All Fields] OR "somalia s"[All Fields]) OR ("south africa"[MeSH Terms] OR ("south"[All Fields] AND "africa"[All Fields]) OR "south africa"[All Fields]) OR ("south sudan"[MeSH Terms] OR ("south"[All Fields] AND "sudan"[All Fields]) OR "south sudan"[All Fields]) OR ("sri lanka"[MeSH Terms] OR ("sri"[All Fields] AND "lanka"[All Fields]) OR "sri lanka"[All Fields]) OR ("saint lucia"[MeSH Terms] OR ("saint"[All Fields] AND "lucia"[All Fields]) OR "saint lucia"[All Fields] OR ("st"[All Fields] AND "lucia"[All Fields]) OR "st lucia"[All Fields]) OR ("saint vincent and the grenadines"[MeSH Terms] OR ("saint"[All Fields] AND "vincent"[All Fields] AND "grenadines"[All Fields]) OR "saint vincent and the grenadines"[All Fields] OR ("st"[All Fields] AND "vincent"[All Fields] AND "grenadines"[All Fields]) OR "st vincent and the grenadines"[All Fields]) OR ("sudan"[MeSH Terms] OR "sudan"[All Fields] OR "sudans"[All Fields] OR "sudan s"[All Fields]) OR ("suriname"[MeSH Terms] OR "suriname"[All Fields] OR "surinam"[All Fields]) OR ("syria"[MeSH Terms] OR "syria"[All Fields] OR ("syrian"[All Fields] AND "arab"[All Fields] AND "republic"[All Fields]) OR "syrian arab republic"[All Fields]) OR ("tajikistan"[MeSH Terms] OR "tajikistan"[All Fields]) OR ("tanzania"[MeSH Terms] OR "tanzania"[All Fields] OR "tanzania s"[All Fields]) OR ("thailand"[MeSH Terms] OR "thailand"[All Fields] OR "thailand s"[All Fields]) OR ("timor leste"[MeSH Terms] OR "timor leste"[All Fields] OR ("timor"[All Fields] AND "leste"[All Fields]) OR "timor leste"[All Fields]) OR ("togo"[MeSH Terms] OR "togo"[All Fields]) OR ("tonga"[MeSH Terms] OR "tonga"[All Fields] OR "tonga s"[All Fields]) OR ("tunisia"[MeSH Terms] OR "tunisia"[All Fields]) OR ("turkey"[MeSH Terms] OR "turkey"[All Fields] OR "turkiye"[All Fields]) OR ("turkmenistan"[MeSH Terms] OR "turkmenistan"[All Fields]) OR ("micronesia"[MeSH Terms] OR "micronesia"[All Fields] OR "tuvalu"[All Fields]) OR ("uganda"[MeSH Terms] OR "uganda"[All Fields] OR "uganda s"[All Fields]) OR ("ukraine"[MeSH Terms] OR "ukraine"[All Fields] OR "ukraine s"[All Fields]) OR ("uzbekistan"[MeSH Terms] OR "uzbekistan"[All Fields]) OR ("vanuatu"[MeSH Terms] OR "vanuatu"[All Fields]) OR (("venezuela"[MeSH Terms] OR "venezuela"[All Fields] OR "venezuela s"[All Fields]) AND "RB"[All Fields]) OR ("vietnam"[MeSH Terms] OR "vietnam"[All Fields] OR "vietnam s"[All Fields]) OR (("middle east"[MeSH Terms] OR ("middle"[All Fields] AND "east"[All Fields]) OR "middle east"[All Fields] OR ("west"[All Fields] AND "bank"[All Fields]) OR "west bank"[All Fields]) AND "Gaza"[All Fields]) OR (("yemen"[MeSH Terms] OR "yemen"[All Fields]) AND "rep"[All Fields]) OR ("zambia"[MeSH Terms] OR "zambia"[All Fields] OR "zambia s"[All Fields]) OR ("zimbabwe"[MeSH Terms] OR "zimbabwe"[All Fields] OR "zimbabwe s"[All Fields])))
